# fMRI Analysis of Brain Network Connectivity during Adolescence. Knowledge of Normotypic Brain Connectivity Patterns May Further Understanding of Aberrant Processes that Cause Psychiatric Illnesses

**DOI:** 10.1523/ENEURO.0275-18.2018

**Published:** 2018-07-25

**Authors:** Rosalind S.E. Carney

**Highlighted Research Paper:** Key Brain Network Nodes Show Differential Cognitive Relevance and Developmental Trajectories during Childhood and Adolescence, by, Knut K. Kolskår, Dag Alnæs, Tobias Kaufmann, Geneviève Richard, Anne-Marthe Sanders, Kristine M. Ulrichsen, Torgeir Moberget, Ole A. Andreassen, Jan E. Nordvik, and Lars T. Westlye

The transition from adolescence to adulthood is a critical period for human brain development. The maturing adolescent brain must adapt to acquire abilities reminiscent of adulthood, including higher cognitive demands, increased emotional regulation, and social adaptation. A protracted period of neuroplasticity over several years enables new synaptic connections to be created and stabilized/removed between brain regions. During this time, gray matter undergoes extensive synaptic pruning, while white matter volume increases, supporting the emergence and specialization of functional networks. Therefore, it is not surprising that several psychiatric illnesses are thought to result from aberrant connections formed during adolescence. To understand how aberrant connectivity occurs, one must first have a thorough understanding of how normotypic connectivity patterns are established.

Advances in brain imaging technology have motivated a vast interest in functional connectivity analyses. Just as commercial airlines use central airports as hubs to concentrate operations and increase efficiency, the brain also employs hubs as locations to integrate widespread neural activity (Miši and Sporns, 2016). These hubs are neural “hot spots,” highly connected nodes that mediate communication within and between large-scale neural networks. The importance of a particular node can be defined by its centrality, a measure of the number and strength of connections an individual node has, relative to the rest of the network.

**Figure 1.**
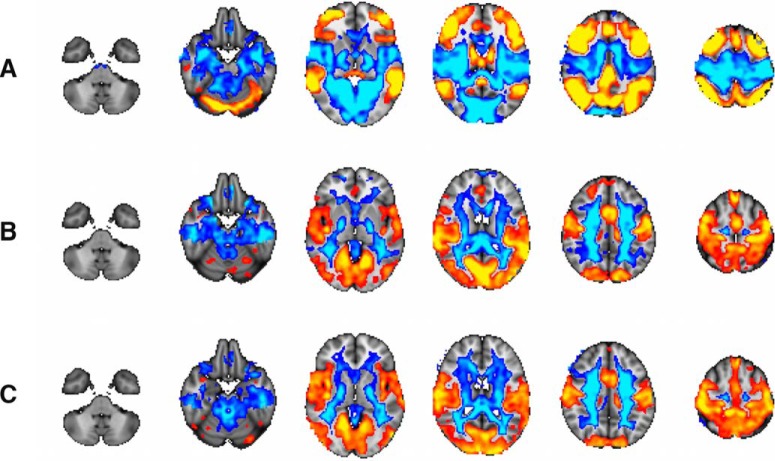
Results from full-brain voxel-wise analysis. ***A***, Significant changes in EC associated with differentiation between task and rest across age span. Age-effect in the working memory task data (***B***) and age-effect in the rest data (***C***; figure modified from Figure 4 in Kolskår et al., 2018).

In their *eNeuro* publication, Kolskår and et al., 2018, analyzed fMRI data from 754 children and adolescents, aged 8–22 years. These data were collected as part of the Philadelphia Neurodevelopmental Cohort, a large-scale study that examined how brain maturation impacts cognitive development and vulnerability to psychiatric illnesses (Satterthwaite et al., 2013). The subjects were recorded during two separate psychological conditions: resting state and a working memory task. Kolskår and colleagues applied a graph-based method, termed eigenvector centrality (EC), which estimates the centrality of every point (voxel) in the brain, reflecting its integration within the entire connectome. After computing brain-wide EC maps for each subject for both psychological conditions, they used a data-driven method (independent component analysis; ICA), to decompose the individual EC maps into several large-scale brain spatial maps reflecting measures of centrality across subjects and psychological conditions. Compared to important prior studies that examined the functional connectivity of fewer nodes (Hwang et al., 2013; Sato et al., 2015), this novel approach allowed Kolskår and colleagues to analyze several spatially independent, large-scale brain networks, including those with activation patterns that have previously been described for sensory, motor, and cognitive functions (Figure 1).

From this complex series of analyses, Kolskår and colleagues report three main findings.

1. Working memory task performance is associated with increased centrality of key nodes within the frontoparietal, cerebellar, and cingulo-opercular networks, and decreased centrality of sensorimotor and thalamic nodes. This shift in hub properties in response to task performance is suggested to support increased efficacy of interactions between relevant nodes while suppressing the activation of task-irrelevant nodes.
2. Age-related centrality differences in cingulo-opercular, visual, and sensorimotor network nodes were observed during both the resting state and task performance. This observation suggests that common neurodevelopmental processes manifest across both of the psychological conditions tested.
3. The centrality of key cingulo-opercular network nodes increased during task performance. Taken together with the age-related differences, this finding highlights the importance of cingulo-opercular hubs in neurocognitive development.

This study provides an increased understanding of normotypic patterns of functional connectivity during adolescent brain development and their implications for cognitive functioning. In particular, the study emphasizes the significance of cingulo-opercular hubs in brain maturation with respect to cognitive development. As recent studies in adults have implicated deviant hub functioning as one of the underlying mechanisms in several psychiatric illnesses (Crossley et al., 2014), it is crucial to identify further the neural correlates during adolescence that may confer an increased risk for such illnesses (Kaufmann et al., 2017). This study by Kolskår and colleagues reflects an important step to support the long-term goal of identifying aberrant connectivity patterns that may underlie functional defects observed in adulthood.
